# Validation of an Automated, End-to-End Metagenomic Sequencing Assay for Agnostic Detection of Respiratory Viruses

**DOI:** 10.1093/infdis/jiae226

**Published:** 2024-05-02

**Authors:** Nick P G Gauthier, Wilson Chan, Kerstin Locher, Duane Smailus, Robin Coope, Marthe Charles, Agatha Jassem, Jennifer Kopetzky, Samuel D Chorlton, Amee R Manges

**Affiliations:** Department of Microbiology and Immunology, University of British Columbia, Vancouver, British Columbia, Canada; School of Population and Public Health, University of British Columbia, Vancouver, British Columbia, Canada; Division of Medical Microbiology, Vancouver General Hospital, Vancouver, British Columbia, Canada; Department of Pathology and Laboratory Medicine, University of British Columbia, Vancouver, British Columbia, Canada; Canada’s Michael Smith Genome Sciences Centre at BC Cancer, Vancouver, British Columbia, Canada; Canada’s Michael Smith Genome Sciences Centre at BC Cancer, Vancouver, British Columbia, Canada; Division of Medical Microbiology, Vancouver General Hospital, Vancouver, British Columbia, Canada; Department of Pathology and Laboratory Medicine, University of British Columbia, Vancouver, British Columbia, Canada; British Columbia Centre for Disease Control, Vancouver, British Columbia, Canada; British Columbia Centre for Disease Control, Vancouver, British Columbia, Canada; BugSeq Bioinformatics Inc, Vancouver, British Columbia, Canada; School of Population and Public Health, University of British Columbia, Vancouver, British Columbia, Canada; British Columbia Centre for Disease Control, Vancouver, British Columbia, Canada

**Keywords:** next-generation sequencing, viral diagnostics, metagenomic, molecular diagnostic

## Abstract

**Background:**

Current molecular diagnostics are limited in the number and type of detectable pathogens. Metagenomic next-generation sequencing (mNGS) is an emerging, and increasingly feasible, pathogen-agnostic diagnostic approach. Translational barriers prohibit the widespread adoption of this technology in clinical laboratories. We validate an end-to-end mNGS assay for detection of respiratory viruses. Our assay is optimized to reduce turnaround time, lower cost per sample, increase throughput, and deploy secure and actionable bioinformatic results.

**Methods:**

We validated our assay using residual nasopharyngeal swab specimens from Vancouver General Hospital (n = 359), which were reverse-transcription polymerase chain reaction positive, or negative for influenza, severe acute respiratory syndrome coronavirus 2, and respiratory syncytial virus. We quantified sample stability, assay precision, the effect of background nucleic acid levels, and analytical limits of detection. Diagnostic performance metrics were estimated.

**Results:**

We report that our mNGS assay is highly precise and semiquantitative, with analytical limits of detection ranging from 10^3^ to 10^4^ copies/mL. Our assay is highly specific (100%) and sensitive (61.9% overall: 86.8%; reverse-transcription polymerase chain reaction cycle threshold < 30). Multiplexing capabilities enable processing of up to 55 specimens simultaneously on an Oxford Nanopore GridION device, with results reported within 12 hours.

**Conclusions:**

This study report outlines the diagnostic performance and feasibility of mNGS for respiratory viral diagnostics, infection control, and public health surveillance. We addressed translational barriers to widespread mNGS adoption.

## Teaser

We optimize and validate an end-to-end metagenomic sequencing assay for detection and characterization of respiratory RNA viruses. We address key translational barriers and highlight the utility of this technology for routine clinical use.

Current standard molecular viral diagnostic assays are cost-effective, highly sensitive, and clinically actionable. However, in some cases direct sequencing of clinical specimens is feasible and may provide added value, including agnostic surveillance for emerging pathogens, diagnosis of infections when conventional testing results are negative, and routine viral diagnostics in situations where multiplex nucleic acid amplification tests would traditionally be ordered [1, 2]. Such multiplex panels are limited, as they detect only those pathogens to which their primers were designed; untargeted or emerging pathogens or those with mutations in primer binding sites will be missed. Metagenomic next-generation sequencing (mNGS) is a pathogen-agnostic diagnostic approach that enables nonspecific detection and characterization of total nucleic acid content directly from clinical specimens.

Several studies have examined the feasibility and utility of mNGS for a range of diagnostic uses, most of which has been focused on diagnosis of pathogens in sterile body fluids, such as cerebrospinal fluid and plasma [3–5]. More recently, several groups, including ours, have demonstrated the feasibility of mNGS for diagnosis of respiratory infections [6–11], as well as for surveillance and infection control applications. Despite advancements in the technical performance of mNGS for viral pathogen detection, several barriers remain that prohibit the translation and widespread adoption of mNGS for routine viral diagnostics [1]. These include high per-sample costs, intricate and nonautomated sample preparation workflows, off-target amplification and reagent contamination, and a lack of standardized and user-friendly bioinformatics pipelines.

In the current study, we optimized, automated, and validated an end-to-end mNGS assay for agnostic detection of viral pathogens in upper respiratory specimens. We used the Oxford Nanopore Technologies (ONT) sequencing platform because the portability, lower capital cost, and ease of use of this platform make it uniquely tailored to clinical and public health laboratory settings. ONT is a single-molecule, long-read sequencing platform, which can perform analysis in real time. This feature makes the ONT platform well suited to infectious disease diagnostic applications, where turnaround time is a key consideration [2, 12]. Our assay, which we have termed “rapid pathogen identification through next-generation sequencing” (RAPID-mNGS), harnesses the Rapid-SMART9N random priming technique [8] and is automated on a liquid handling robot to increase assay precision, reduce turnaround time, increase throughput, and decrease per-sample cost.

Bioinformatic analysis was performed using BugSeq, a rapid, scalable, and user-friendly, cloud-based pipeline [13, 14]. Analytical and clinical validation of the RAPID-mNGS assay was performed on nasopharyngeal swab (NPS) specimens that were either negative or positive for severe acute respiratory syndrome coronavirus 2 (SARS-CoV-2), respiratory syncytial virus (RSV), and influenza A (target RNA viruses for this study) and were collected from Vancouver General Hospital and the British Columbia Centre for Disease Control during the 2022–2023 respiratory virus season.

## METHODS

### RAPID-mNGS Assay Description

**Figure 1.**
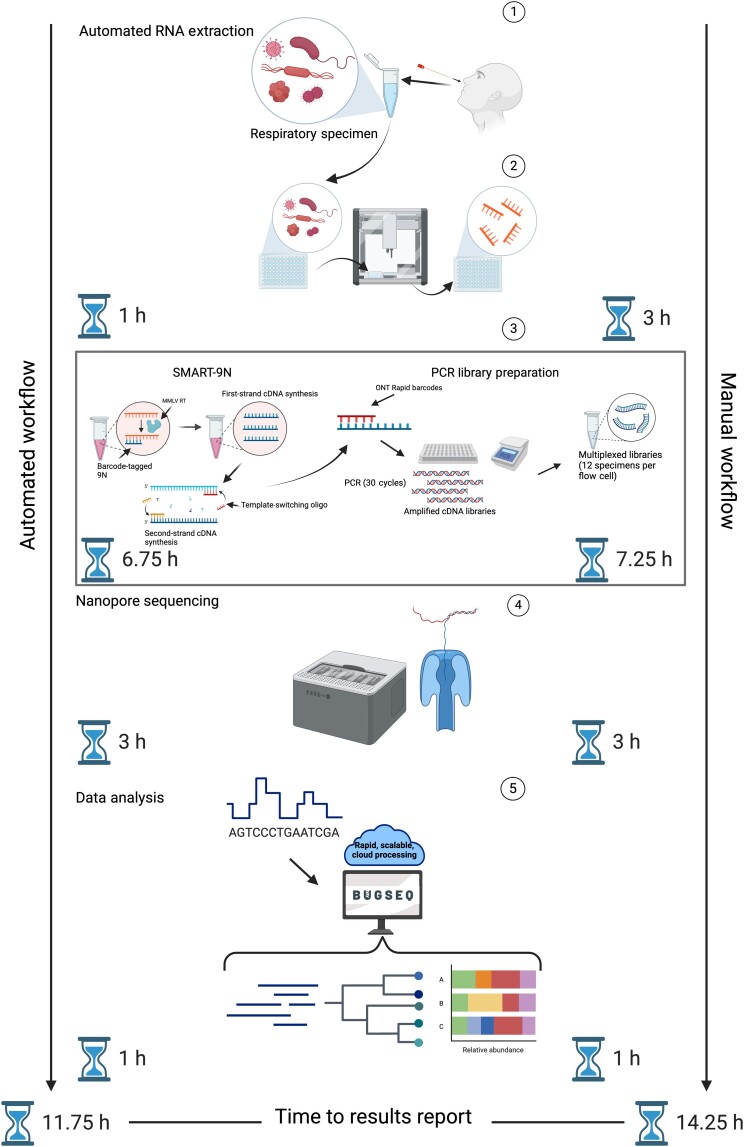
Rapid pathogen identification through next-generation sequencing assay workflow. Arrow and hourglass icons on the left-hand side represent times required for each protocol step for the automated workflow. The right-hand side represents times required for the manual workflow. Abbreviations: cDNA, complementary DNA; MMLV, Moloney Murine Leukemia Virus; ONT, Oxford Nanopore Technologies; PCR, polymerase chain reaction.

The optimized RAPID-mNGS assay was automated using a Hamilton Microlab NIMBUS96 liquid handling robot (extraction, reverse-transcription [RT], library preparation). An overview of the RAPID-mNGS assay, along with time savings for the automated workflow compared to manual specimen processing, is shown in Figure 1. We observed a high level of concordance in viral read counts for the manual and automated protocols (Supplementary Figure 1 and Supplementary Methods). RNA extraction began with 200 μL of up to 55 specimens (plus 1 no-template control per flow cell) that were spun down at 16 000*g* for 3 minutes. The 135 μL of each specimen was added to a Kingfisher MagMax deep-well plate containing 270 μL of lysis buffer. MS2 bacteriophage (American Type Culture Collection 15997-B1) was spiked into each specimen (5 × 10^5^ copies) as an internal process control. Specimens were incubated for 10 minutes, followed by the addition of 400 μL of EvoPure magnetic bead mix (Aline Biosciences), and placed on the deck of the NIMBUS for RNA extraction, as described elsewhere [15], with an additional ethanol wash and elution into 100 μL of nuclease-free water.

Several host nucleic acid depletion approaches were assessed, including depletion of abundant sequences through hybridization (DASH), a Cas9-based host depletion approach, and ONT adaptive sampling, a tool used to deplete reads aligning to the host genome while sequencing in real time. However, we did not observe significant viral enrichment from either of these methods (Supplementary Figure 2 and Supplementary Methods). Random RT was performed as described elsewhere [8], and libraries were amplified with 30 polymerase chain reaction (PCR) cycles with index-attached PCR primers (SQK-RPB004 and RLB01-12; ONT). Amplified libraries underwent a 0.6× PCRClean DX (Aline Biosciences) library clean-up, followed by quantification using a Qubit 4 fluorometer (Thermo Fisher Scientific) and equimolar pooling of 12 specimens per flow cell. Pooled libraries were sequenced on an ONT GridION sequencing device (R9.4.1 flow cells) for up to 72 hours; however, sequence data were processed after 3 hours of sequencing to reduce assay turnaround time (Figure 1).

### Bioinformatic Analysis & Data Visualization

Raw sequencing data were filtered to remove host reads using minimap2 [16] alignment against the human genome (GRCh38). The remaining reads were uploaded to the secure BugSeq cloud-based analysis portal (version 4.0). The BugSeq workflow has been described elsewhere [13, 14]. Detection of 1 read was used as the threshold to call a virus as present. Failure to detect MS2 reads constituted a failed library. Barcode cross-talk correction, which removed 0.017% (0.2% cross-talk rate per flow cell divided by 12 samples per flow cell) of the total number of reads at each taxonomic rank on a given flow cell from each sample, was performed to maintain assay specificity, as described elsewhere [7, 17].

To validate the cross-talk correction, a set of 3 flow cells were sequenced, each containing ≥1 SARS-CoV-2 specimen with high viral load (RT-PCR cycle threshold [C_t_] <15) (21 SARS-CoV-2–positive and 12 negative specimens). mNGS data were analyzed after 3 hours of sequencing with and without cross-talk correction. Viral and MS2 bacteriophage read counts were summarized from annotated tables generated by BugSeq and imported into RStudio (R Version 4.3.1) for statistical analysis and data visualization using ggplot2 [18]. Sensitivity and specificity were estimated for each virus and overall. Sensitivity analyses were performed to quantify diagnostic performance by sequencing duration. Workflow diagrams were designed using Biorender software (biorender.com).

### Analytical Validation

Host nucleic acid quantification was performed using RT-PCR against the host RnaseP gene. Viral nucleic acid loads were quantified for NPS specimens positive for influenza A, SARS-CoV-2, or RSV (see Supplementary Tables 1 and 2). An additional set of 21 parainfluenza virus–positive (n = 10) and Enterovirus-positive (n = 11) specimens by the qualitative NxTag Respiratory Pathogen Panel (Luminex) from the British Columbia Centre for Disease Control public health laboratory were sequenced for demonstration of assay inclusivity. RT-PCR C_t_ values were converted to genome copies per milliliter using a standard curve for both viral and host nucleic acid loads. Assay stability was quantified by subjecting a set of 4 NPS specimens—positive for SARS-CoV-2 by routine RT-PCR testing and spanning a range of viral titers—to varying storage conditions (−80°C, −20°C, and 4°C) and durations (0, 45, and 90 days). Virus stability was evaluated by assessment of log-fold change in viral reads from baseline. Assay precision was estimated by replicate testing of 11 NPS specimens (influenza A [n = 2], SARS-CoV-2 [n = 3], RSV [n = 4], negative [n = 2]) on successive days with distinct reagent lots and 2 instrument operators.

**Figure 2.**
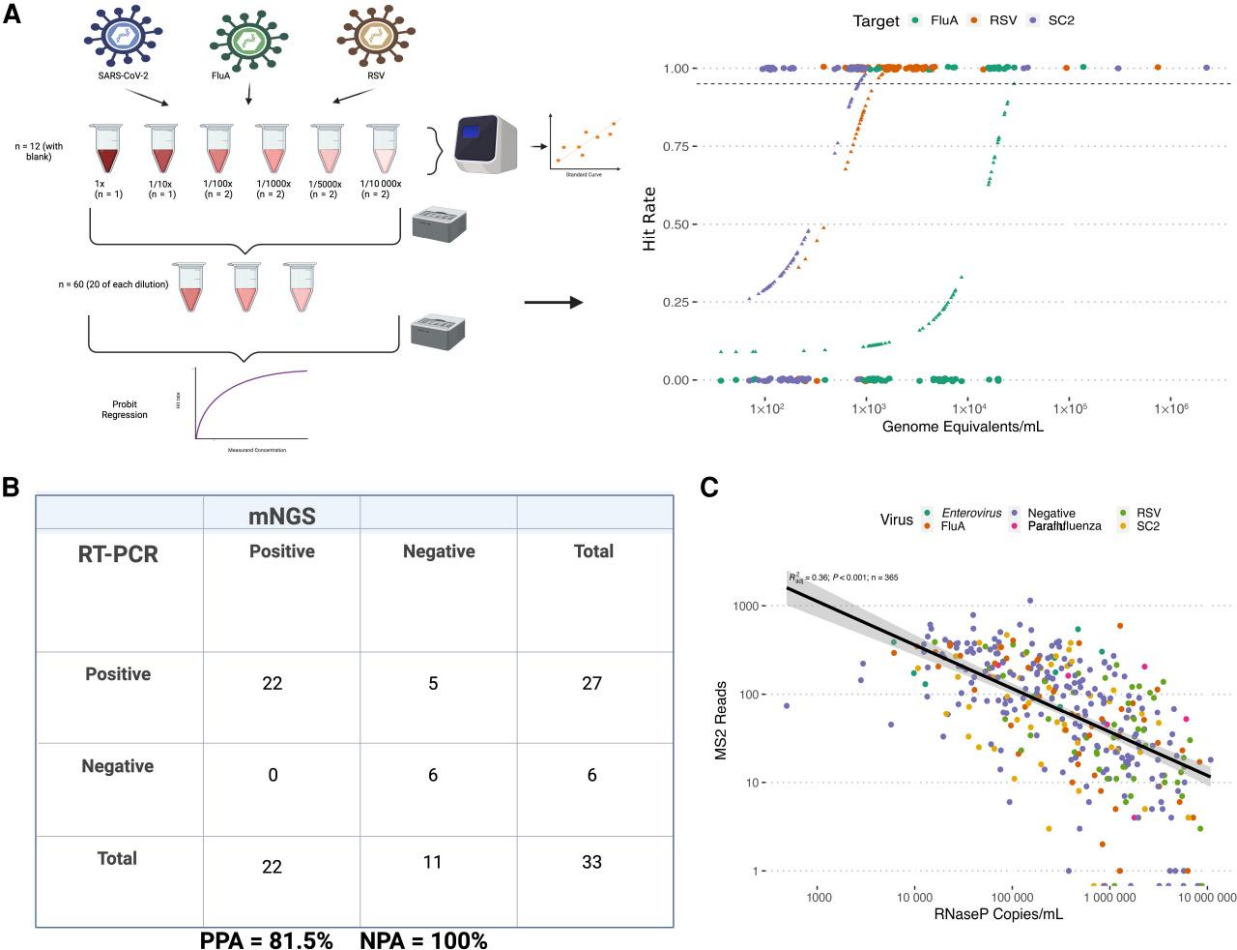
*A*, Overview of the limits of detection (LOD) study design. Specimens underwent a preliminary dilution series and were sequenced, following which a set of 3 dilutions flanking the hypothesized LOD were chosen for replicate testing (n = 20 of each dilution for each virus). Probit regression models were fit for each virus: influenza A (FluA; *green (right)*), respiratory syncytial virus (RSV; *orange (middle)*), and severe acute respiratory syndrome coronavirus 2 (SARS-CoV-2; *purple (left)*). Horizontal dotted line represents the concentration of virus with a 95% detection probability. *B*, Positive percent agreement (PPA) and negative percent agreement (NPA) for the set of 11 specimens used for the assay precision estimates, which were divided into 3 aliquots and sequenced on subsequent days. *C*, Linear model of MS2 bacteriophage read counts against host nucleic acid loads, as determined from RnaseP gene quantitative reverse-transcription polymerase chain reaction (RT-PCR). Absolute viral loads were determined using a standard curve. Abbreviations: mNGS, metagenomic next-generation sequencing; SC2, severe respiratory syndrome coronavirus 2.

RT was performed on each replicate set, and complementary DNA was frozen at −80°C before library preparation to ensure that each aliquot was subjected to a single freeze-thaw cycle. Library preparation was performed on 3 separate days. Positive and negative percent agreements were calculated, along with the coefficient of variation for MS2 reads across each set of replicate sequencing runs. Analytical limits of detection (LOD) studies were designed based on (Clinical and Laboratory Standards Institute) EP17-A2 guidelines and published recommendations for mNGS assay validation [19], using serial dilutions of NPS specimens with high viral loads (RT-PCR C_t_ <20) (n = 72 total sample dilution replicates, for each virus bracketing the expected LOD) (Figure 2*A*). Viral nucleic acid loads were quantified for each replicate, as outlined above. LOD were calculated for each virus using probit regression.

### Clinical Validation

Residual, deidentified NPS specimens immersed in viral transport medium were collected in coordination with the (Diagnostic Accreditation Program-accredited) Division of Medical Microbiology at Vancouver General Hospital for validation of the RAPID-mNGS assay. NPS specimen RT-PCR C_t_ value were captured (Panther Fusion Flu A/B/RSV Assay and SARS-CoV-2 Assay [Hologic] or Xpert Xpress CoV-2/Flu/RSV plus Assay [Cepheid]). Study specimens were collected on a weekly basis, transferred, and stored at −80°C until RAPID-mNGS sample processing. Specimens were sequenced in random order to reduce the potential for batch effects. Specimens with discordant results between the reference-standard comparator assay and the RAPID-mNGS assay and specimens with incidental viral pathogens detected that were not tested for in the original diagnostic panel were retested at Vancouver General Hospital using the BioFire Respiratory Pathogen Panel 2.1 (bioMérieux) when possible.

### Ethical Considerations and Data Availability

University of British Columbia research ethics board approval was obtained (H22-0199). Patient consent was not obtained, as residual and deidentified specimens were used. Nonhost sequence data are available at National Center for Biotechnology Information BiopProject accession number PRJNA1092681.

## RESULTS

### Analytical Validation

Analytical LOD (95% hit probability) were determined for influenza A, SARS-CoV-2, and RSV. We report LOD of 836 copies/mL, 1219 copies/mL, and 28 709 copies/mL for SARS-CoV-2, RSV, and influenza A, respectively (Figure 2*A*). We detected parainfluenza virus reads in 7 of 10 specimens and *Enterovirus* in 7 of 11, indicating inclusivity. We validated our barcode cross-talk correction threshold. Cross-contamination was estimated to be 0.0081%, 0.0034%, and 0.015% for each of the 3 flow cells (Supplementary Table 3), indicating that the previously established cross-talk correction threshold [7] maintains a high level of specificity without sacrificing sensitivity.

We observed no loss in SARS-CoV-2 read recovery for any sample storage temperature for up to 90 days (Supplementary Figure 3A). SARS-CoV-2 reads were most abundant in the −80°C and −20°C storage temperatures. We did observe a decrease in host reads from 0 to 90 days across all specimens and storage conditions, suggesting that viral particles are more resistant to RNA degradation in viral transport medium than host RNA (Supplementary Figure 3B). MS2 read distributions did not differ significantly between 3 subsequent sequencing runs; a between-run coefficient of variation was estimated to be 22.9% (Supplementary Figure 4). The percent positive agreement was 81.5% and percent negative agreement was 100% for the RAPID-mNGS assay, compared with reference-standard assay results (Figure 2*B*). A linear model was fit to model MS2 reads against host nucleic acid loads, and we observed a modest inverse relationship between MS2 reads recovered and host nucleic acid background levels (*R*^2^ = 0.36; Figure 2*C*).

A blinded, novel pathogen exercise was performed to demonstrate the utility of the RAPID-mNGS assay for detection of emerging pathogens (Supplementary Methods). The 12 negative specimens were correctly identified by our team. Our team successfully identified the viral family for the influenza samples (Orthomyxoviridae) and the genus for the RSV samples (*Orthopneumovirus*), without overclassifying to other Orthomyxoviridae or *Orthopneumovirus* species. Repeating this exercise with the RefSeq database instead of BugSeq's curated database resulted in 8 errors from taxonomic misannotation or RefSeq genome contamination.

### Clinical Validation

Study NPS specimens (n = 359) were positive for influenza A (n = 53), RSV (n = 57), or SARS-CoV-2 (n = 51) by routine diagnostic RT-PCR testing, or they were negative for any of the target viruses listed above (n = 198). MS2 process control reads were detected in 347 of 359 specimens (96.7%). We correctly identified viral pathogens in 96 of 155 RT-PCR–positive specimens overall (61.9% [95% confidence interval, 53.8%–69.6%]) and in 92 of 106 (86.8% [78.8%–92.6%]) for specimens with an RT-PCR C_t_ <30 after 3 hours of sequencing. The overall sensitivity was lower for specimens with low viral loads (8.2% [4 of 49]; RT-PCR C_t_ >30). We report 100% specificity overall (192 of 192 [95% confidence interval, 98.1%–100%]). We confirmed the absence of viral read contamination in our negative controls across all sequenced flow cells. Per-sample sequence data summaries are available in the Supplementary Data.

**Figure 3.**
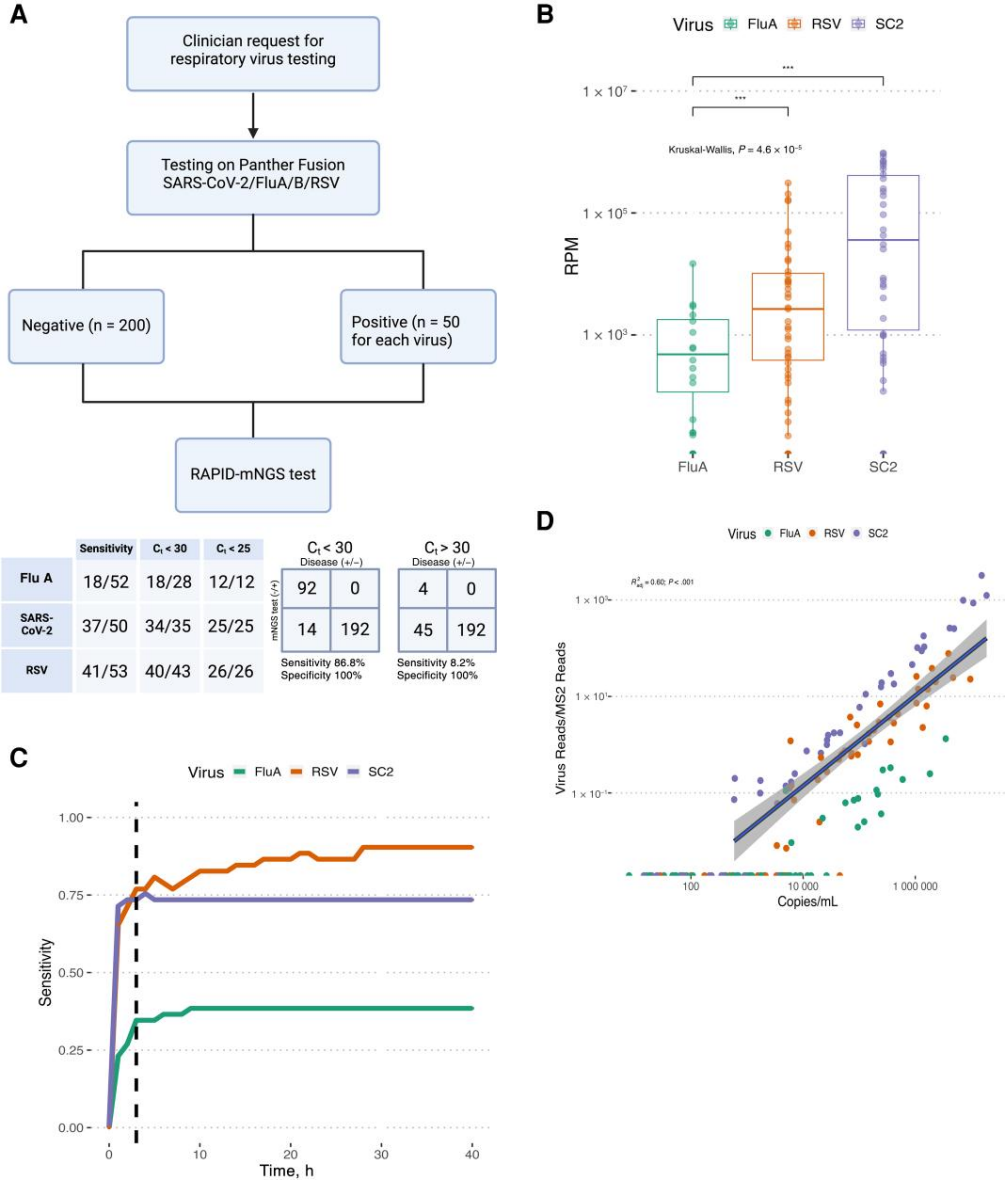
*A*, Overview of the clinical validation study workflow, as well as rapid pathogen identification through next-generation sequencing (RAPID-mNGS) assay sensitivity and specificity estimates stratified by viral pathogen and viral load. *B*, Distribution of normalized viral read counts (reads per million reads [RPM]) for reverse-transcription polymerase chain reaction (RT-PCR)–positive viral specimens used in the clinical validation study. *C*, Sensitivity analysis displaying changes in cross-talk­–corrected sensitivity estimates for each additional hour of sequencing duration (up to 40 hours). *D*, Relationship between normalized viral reads (total viral reads/total MS2 reads) and absolute viral load determined from RT-PCR. Colored dots represent specimens positive for either influenza A (FluA), respiratory syncytial virus (RSV), or severe acute respiratory syndrome coronavirus 2 (SARS-CoV-2). Abbreviations: C_t_, cycle threshold; SC2, severe respiratory syndrome coronavirus 2.

Virus-specific sensitivity estimates were aligned with analytical LOD (Figure 3*A*); the RAPID-mNGS assay was more sensitive for RSV and SARS-CoV-2 than for influenza A. Sensitivity estimates were correlated with normalized viral reads (reads per million reads) recovered in mNGS data (Figure 3*B*). A sensitivity analysis to assess the impact of sequencing duration on overall assay sensitivity (Figure 3*C*) indicates that a 3-hour sequencing duration maintains high sensitivity while minimizing assay turnaround time; however, accumulation of viral sequencing reads did not plateau until about 20 hours of sequencing (Supplementary Figure 5), reflecting the sequencing capacity of the R9.4.1 flow cell. Finally, we observed a moderately strong linear relationship between the ratio of viral reads to MS2 reads and absolute RT-PCR–based viral load (Figure 3*D*). This ratio was more strongly correlated with viral load than viral reads per million reads, highlighting that the RAPID-mNGS assay can be used reliably for viral quantitation, although virus-specific calibration curves would improve the accuracy of quantification estimates (virus-specific *R*^2^ = 0.51 [influenza A], 0.80 [RSV], and 0.95 [SARS-CoV-2]). We were also able to assign 78.9% (75 of 95) of mNGS-positive SARS-CoV-2, RSV, and influenza A specimens to the subtype/lineage level (Supplementary Table 4).

We report the detection of several nontarget viral pathogens for which specimens were not initially screened (Table 1). Overall, we detected with RAPID-mNGS ≥1 nontarget viral pathogen in 14 of 347 specimens (4%). Of these 14 specimens, 5 (35.7%) were also RT-PCR positive for SARS-CoV-2, RSV, or influenza A. Viral infection was confirmed for 10 of 10 tested specimens (100%) using the BioFire Respiratory Pathogen Panel 2.1. For the remaining 4 specimens, either there was no remaining specimen after RAPID-mNGS testing or a reference-standard test was not available.

**Table 1.** Incidental Detection of Viral Pathogens in 14 Specimens

Virus	Mapped Reads	Average Primary Alignment, bp	Average % Identity	Confirmatory Testing Performed
Human metapneumovirus	65	4237	95.96	Yes
	26	1218	94.89	Yes
	9	3104	96.86	Yes
Human β-herpesvirus 6B	7	2991	97.02	NA
Human coronavirus HKU1	1	1869	92.82	Yes
Human coronavirus OC43	28	1167	97.37	Yes
	234	4246	96.43	Yes
	555	3721	96.66	No
Human parvovirus B19	55	3263	95.98	NA
Human mastadenovirus B	41	3965	96.57	Yes
Human papillomavirus (type 129)	1	1330	96.84	No
Parainfluenza virus 2	7	3925	96.44	Yes
Parainfluenza virus 3	499	3597	93.73	Yes
Rhinovirus C	3	5103	86.66	Yes

Abbreviations: bp, base pairs; NA, not available.

## DISCUSSION

This report describes the largest and most comprehensive validation of an end-to-end mNGS assay for detection of respiratory viral pathogens to our knowledge. we demonstrated that the RAPID-mNGS assay is sensitive, highly specific, and agnostic for detection of viral pathogens and may soon be adapted for use as a diagnostic, surveillance, pandemic preparedness, and infection control tool. However, the observed low sensitivity for more weakly positive specimens limits the immediate use of this assay for diagnostic screening. We addressed many translational barriers [1] to assay implementation and optimized our assay for speed (results within 12 hours), throughput (processing of up to 55 specimens simultaneously), reliability, inclusivity, and performance. We report that when multiplexing 12 specimens per flow cell, the per-sample cost of the RAPID-mNGS assay is approximately $100.

The overall sensitivity (RT-PCR C_t_ <30) and specificity for the target viruses using RAPID-mNGS were 86.8% and 100%, respectively, although the sensitivity was lower for specimens with lower target viral loads. The entire protocol from nucleic acid extraction to result could be achieved in 12 hours, with assay automation leading to a 2.5-hour reduction in hands-on time and enabling simultaneous processing of 55 specimens using the ONT GridION. The assay was robust to storage duration and temperature and did not vary substantially by operator or reagent lots. We reported incidental detection of nontarget viral pathogens in 4% of specimens. While many of these were specimens negative for SARS-CoV-2, RSV, and influenza A, we detected several potential coinfections, including in 1 specimen where reads from 3 distinct viruses (RSV, human parvovirus B19, and human coronavirus OC43) were detected, despite the absence of these viruses on the flow cell or in negative controls. Original diagnostic results for this specimen reported only RSV. The incidental detection of viral pathogens in “negative” specimens emphasizes the potential utility of an agnostic diagnostic assay in clinical practice.

Sensitivity analyses revealed that the 3-hour sequencing duration allows for an optimal balance of turnaround time and sensitivity. However, viral read accumulation continued until plateauing at approximately 20 hours of sequencing, which reflects flow cell capacity. The ability for users to analyze sequencing data continuously is a unique feature of the ONT sequencing platform. Short sequencing durations (approximately 3 hours) may be sufficient for detection of emerging viral pathogens, during infection control practice, or in cases in which a physician indicates that turnaround time is critical. Alternatively, analysis of data following extended sequencing durations may be useful for public health surveillance where high sensitivity and/or generation of high-coverage genomes is a priority and timing is less critical. Furthermore, we validated the assay for multiplexing of 12 specimens per flow cell. While this reduced the per-sample cost of the assay, there is a trade-off between per-sample cost and sensitivity. Improvements in sensitivity may be possible by reducing the number of multiplexed samples or increasing sequencing depth by using a higher-throughput sequencing instrument (eg, PromethION 2 Solo), but this would increase per-sample testing costs.

We observed a limited effect of host background nucleic acid levels on MS2 read counts. While there was a significant linear relationship, the strength of the correlation was low, indicating that host nucleic acid background levels explain only a modest amount of the variation in observed MS2 reads. Inclusion of an internal process control when validating and implementing mNGS assays is important for identifying failed libraries and can be used to normalize viral read counts to estimate viral loads from mNGS data with high concordance to RT-PCR quantification results.

Development of user-friendly and reliable bioinformatics pipelines is key to the translation of mNGS-based diagnostics. We demonstrated that BugSeq can provide interpretable results based on mNGS data in <1 hour. We also showed that we can accurately classify viral pathogen presence/absence in upper respiratory specimens with high specificity using a conservative barcode cross-talk correction [7, 17]. Our experience with this platform highlighted the value of managed information technology infrastructure, automation, and version control; for the latter, we were able to lock down the execution code and database after assay optimization to ensure reproducibility for analytical and clinical validation. We also observed the importance of database curation when validating an assay for novel pathogen detection.

This study has several limitations. First, the use of residual NPS specimens limited our ability to evaluate the impact of patient-specific factors on the performance of the RAPID-mNGS assay. We observed low analytical LOD for RSV and SARS-CoV-2; however, the LOD for influenza A was an order of magnitude higher, limiting the immediate usage of this assay for diagnostic screening purposes. Despite this result, the estimated LOD for influenza A was still below typical influenza respiratory viral loads [20]. However, further exploration into sample pretreatment [21] or other host nucleic acid depletion strategies may be required to improve influenza virus sensitivity [22]. Inclusivity testing of the RAPID-mNGS assay on additional specimens positive for parainfluenza virus and *Enterovirus,* as well as incidental detection of other respiratory viruses, suggest that the higher influenza A LOD and lower sensitivity is likely an isolated issue. However, the LOD results stress the importance of performing extensive validation of any mNGS assay against a panel of organisms reflective of the intended pathogen targets.

Assessment of the RAPID-mNGS assay with diverse specimen types, as well as evaluation of its diagnostic performance for bacterial and fungal pathogens, is necessary to determine the extent and scope of its clinical use. Finally, prospective studies or clinical impact trials are also needed to determine the added value of the assay for clinical diagnostics and impact as well as public health applications.

We report a rapid and valid mNGS assay for the diagnosis and characterization of respiratory viral pathogens. We addressed many barriers to translation of the assay and demonstrated that this agnostic diagnostic assay could have immediate utility for viral diagnostics, infection control, and public health surveillance.

## Supplementary Data

Supplementary materials are available at *The Journal of Infectious Diseases* online (http://jid.oxfordjournals.org/). Supplementary materials consist of data provided by the author that are published to benefit the reader. The posted materials are not copyedited. The contents of all supplementary data are the sole responsibility of the authors. Questions or messages regarding errors should be addressed to the author.

## Supplementary Material

jiae226_Supplementary_Data

## Notes

***Acknowledgments.*** We acknowledge Kingsley Gunadasa and Ron Chow for assistance in preparing specimens for testing, as well as John Tyson for consultation regarding assay optimization and troubleshooting.

***Author contributions.*** K. L., M. C., and A. J. contributed to study specimen collection and data curation. N. P. G. G. and A. R. M. obtained institutional ethical approval. N. P. G. G. and W. C. performed sequencing and viral load quantification of specimens. D. S. and R. C. designed and implemented laboratory automation protocols. N. P. G. G. and S. D. C performed data analysis. J. K. assisted in assay optimization and troubleshooting. All authors contributed to the design of this study and reviewed and edited the manuscript.

***Data availability.*** Data are available in the Supplementary Materials. The automated RAPID-mNGS method package file for the Hamilton NIMBUS96 liquid handling robot is available at 10.5281/zenodo.10913327.

***Financial support*.** This work was supported by the Biomedical Advanced Research and Development Authority, Administration for Strategic Preparedness and Response, Department of Health and Human Services (contract 75A50122C00027) and the Canadian Institute for Health Research (Canada Graduate Scholarship–doctoral fellowship to N. P. G. G.)
