# Supplementary material for: Validation of an Automated, End-to-End Metagenomic Sequencing Assay for Agnostic Detection of Respiratory Viruses
Source: J Infect Dis. 2024 May 2;230(6):e1245–53. doi: 10.1093/infdis/jiae226 (PMC11646614; doi:10.1093/infdis/jiae226)
Supplement: jiae226_Supplementary_Data [file jiae226_supplementary_data.zip › RAPID-mNGS_JID_Supplementary_Methods_MAR28_2024.docx]

**Supplementary materials**

**Supplementary methods**

*Manual sample processing*

Manual RNA extraction was performed as previously described using the Qiagen QIamp Viral RNA kit (1). While we did not observe significant enrichment in viral reads through DNase treatment and RNA concentration (Data not shown), initial assay optimization included TURBO DNase treatment and RNA concentration protocols that were performed as previously described (1,2).

*Assessment of host depletion approaches*

We assessed several host depletion approaches including, depletion of abundant sequences through hybridization (DASH); a Cas9-based host depletion approach (3). DASH was performed on post-PCR amplified libraries prior to sample pooling. Incubation times were varied from 0.5hr-2hr and compared against a no DASH control. ONT adaptive sampling; an *in-silico* tool that was used to deplete reads aligning to the host genome in real-time was also assessed as a host depletion approach. We evaluated this method using contrived samples with 10^4^ SARS-CoV-2 culture genome copies spiked-in. Half of the sequencing channels for a given flowcell had adaptive sampling enables (yes) and half of the channels had adaptive sampling switched off (no).

*Novel pathogen exercise*

A blinded, novel pathogen exercise was performed. We randomly sequenced 12 negative samples and 12 samples with either RSV, Influenza A or Influenza B; we analyzed the sequencing data using a reference database with all records of those viral species removed. Three databases were created, each omitting either Influenza A, Influenza B or RSV. Twelve positive and 12 negative samples were sequenced and analyzed in random order. N.P.G.G, W.C, and A.R.M were returned assay reports with masked sample identifiers, and had to determine which samples had a virus, as well as the most specific taxonomic label for that virus.

**Supplementary References**

1. 1. Gauthier NPG, Nelson C, Bonsall MB, Locher K, Charles M, MacDonald C, et al. Nanopore metagenomic sequencing for detection and characterization of SARS-CoV-2 in clinical samples. PLoS One [Internet]. 2021;16(11):e0259712. Available from: http://dx.doi.org/10.1371/journal.pone.0259712

2. 2. Claro IM, Ramundo MS, Coletti TM, Da Silva CAM, Valenca IN, Candido DS, et al. Rapid viral metagenomics using SMART-9N amplification and nanopore sequencing. Wellcome Open Research 2021 6:241 [Internet]. 2021 Sep 20 [cited 2022 Oct 11];6:241. Available from: https://wellcomeopenresearch.org/articles/6-241

3. 3. Gu W, Crawford ED, O’Donovan BD, Wilson MR, Chow ED, Retallack H, et al. Depletion of Abundant Sequences by Hybridization (DASH): Using Cas9 to remove unwanted high-abundance species in sequencing libraries and molecular counting applications. Genome Biol. 2016;17(1):1–13.
