# Supplementary material for: Validation of an Automated, End-to-End Metagenomic Sequencing Assay for Agnostic Detection of Respiratory Viruses
Source: J Infect Dis. 2024 May 2;230(6):e1245–53. doi: 10.1093/infdis/jiae226 (PMC11646614; doi:10.1093/infdis/jiae226)
Supplement: jiae226_Supplementary_Data [file jiae226_supplementary_data.zip › Supplementary_Figure_1.docx]

**
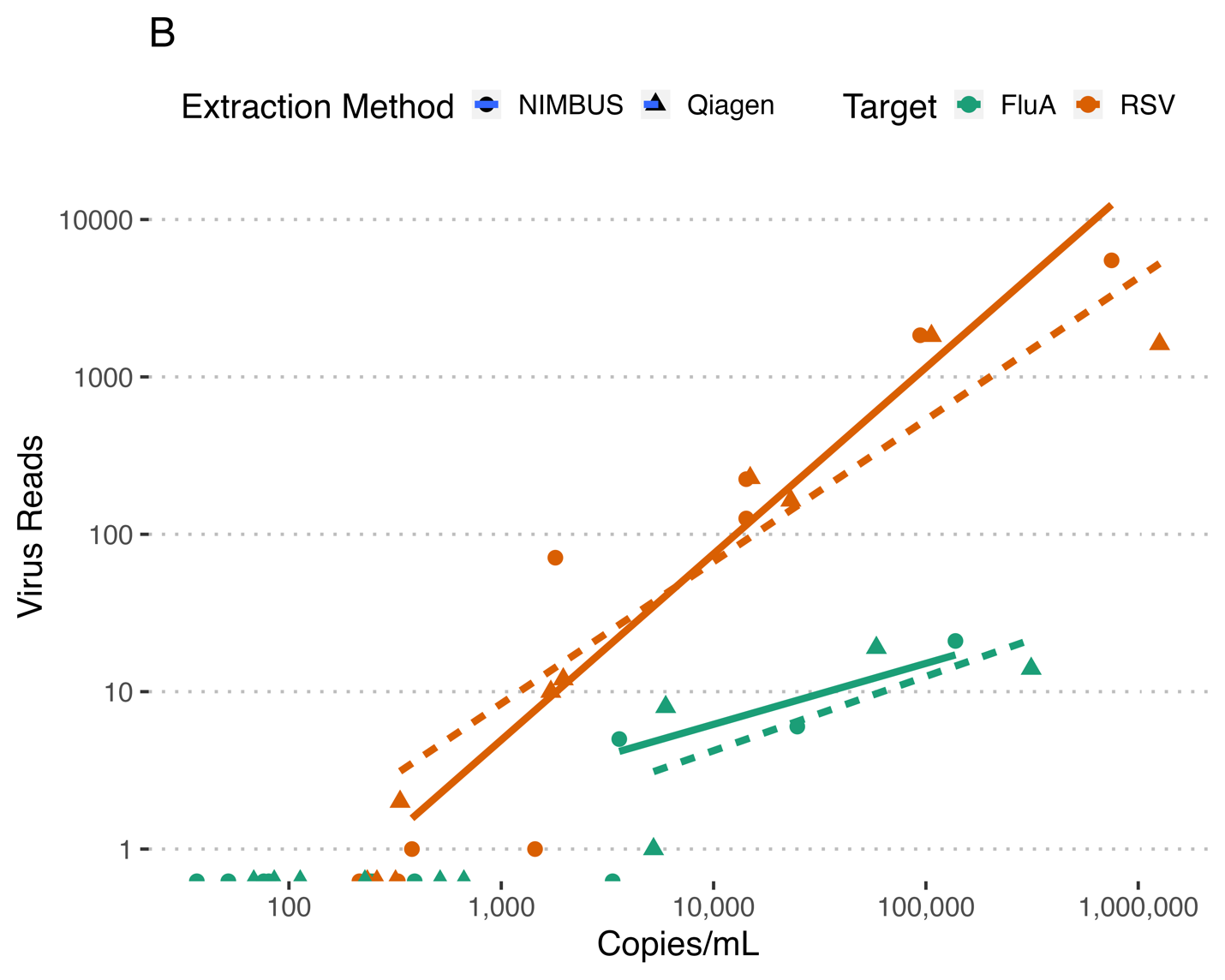
**

**Supplementary Figure 1.** Comparison of viral read counts for serial dilutions of Influenza A (Green) and RSV (Orange) positive nasopharyngeal swab specimens for the manual Qiagen QIAmp Viral RNA extraction kit (Dashed lines and triangles) compared to the automated NIMBUS RNA extraction protocol (Solid lines and circles).
