# Supplementary material for: Validation of an Automated, End-to-End Metagenomic Sequencing Assay for Agnostic Detection of Respiratory Viruses
Source: J Infect Dis. 2024 May 2;230(6):e1245–53. doi: 10.1093/infdis/jiae226 (PMC11646614; doi:10.1093/infdis/jiae226)
Supplement: jiae226_Supplementary_Data [file jiae226_supplementary_data.zip › Supplementary_Figure_2.docx]

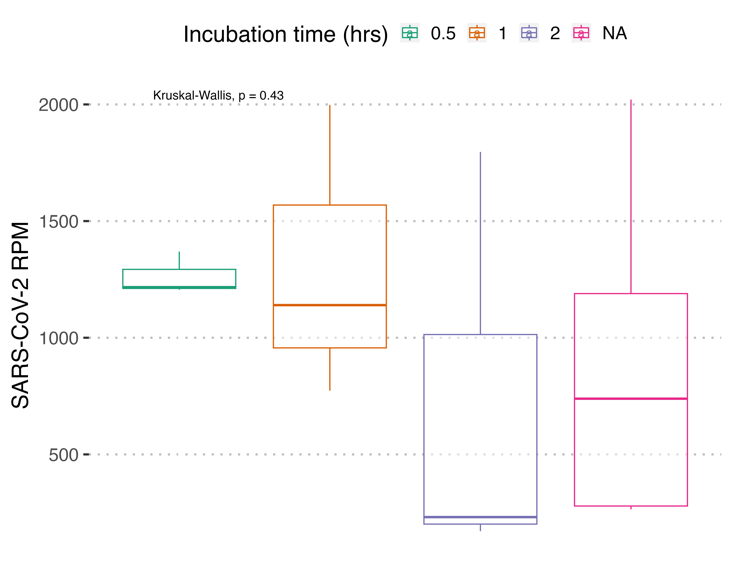

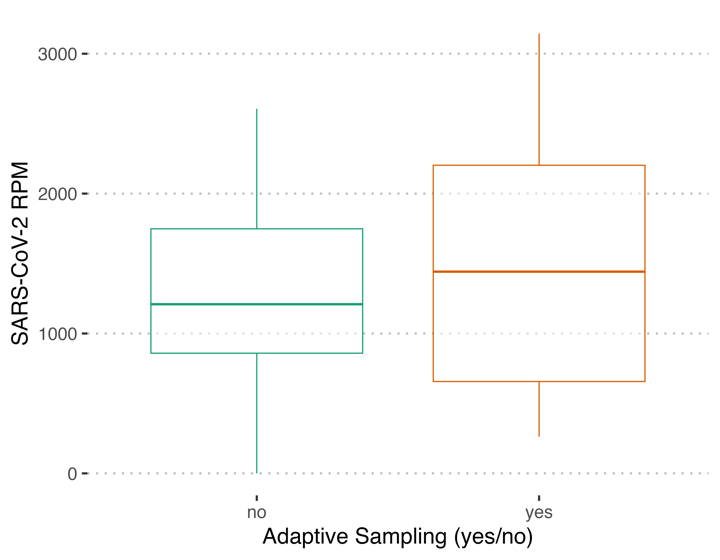


**B**

**A**

**Supplementary Figure 2.** Summary of viral enrichment (RPM; reads per million reads) for SARS-CoV-2 positive nasopharyngeal swab specimens. (A) Assessment of depletion of abundant sequences through hybridization (DASH); a Cas9-based host depletion approach (3). DASH incubation times were varied from 0.5hr-2hr and compared against a no DASH control (NA). (B) Assessment of ONT adaptive sampling; an *in-silico* tool that was used to deplete reads aligning to the host genome in real-time. Half of the sequencing channels for a given flowcell had adaptive sampling enables (yes) and half of the channels had adaptive sampling switched off (no).
