# Supplementary material for: Validation of an Automated, End-to-End Metagenomic Sequencing Assay for Agnostic Detection of Respiratory Viruses
Source: J Infect Dis. 2024 May 2;230(6):e1245–53. doi: 10.1093/infdis/jiae226 (PMC11646614; doi:10.1093/infdis/jiae226)
Supplement: jiae226_Supplementary_Data [file jiae226_supplementary_data.zip › Supplementary_Figure_3.docx]

**A**

**B**


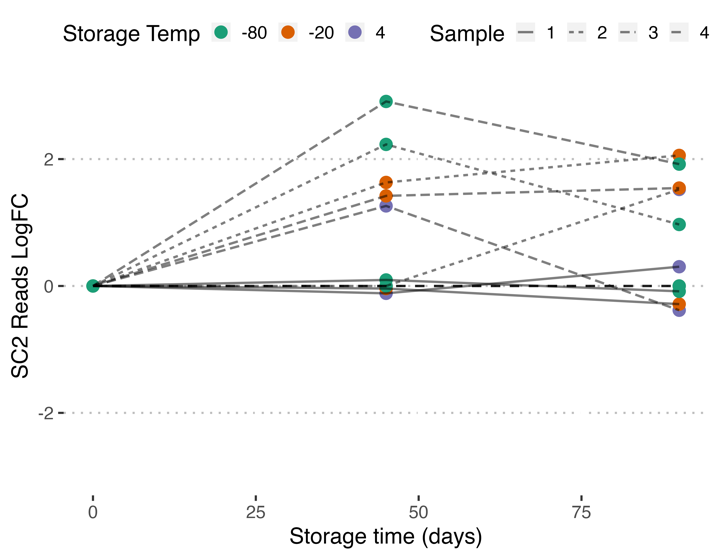

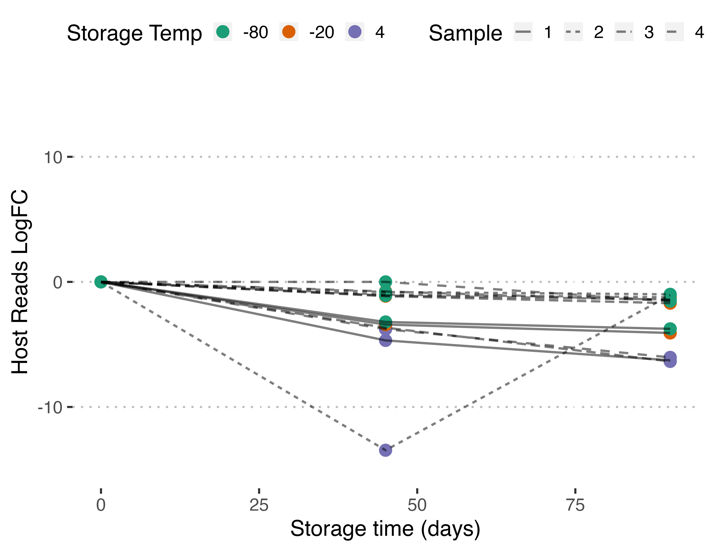


**Supplementary Figure 3.** (A) Log-fold change in normalized SARS-CoV-2 reads (RPM) from baseline to 90-days for aliquots of four SARS-CoV-2 positive nasopharyngeal swab specimens (dashed lines) across three storage conditions. (B) Log-fold change in normalized host reads (RPM) from baseline to 90-days for aliquots of four SARS-CoV-2 positive nasopharyngeal swab specimens (dashed lines) across three storage conditions.
