# Supplementary material for: Validation of an Automated, End-to-End Metagenomic Sequencing Assay for Agnostic Detection of Respiratory Viruses
Source: J Infect Dis. 2024 May 2;230(6):e1245–53. doi: 10.1093/infdis/jiae226 (PMC11646614; doi:10.1093/infdis/jiae226)
Supplement: jiae226_Supplementary_Data [file jiae226_supplementary_data.zip › Supplementary_Figure_4.docx]

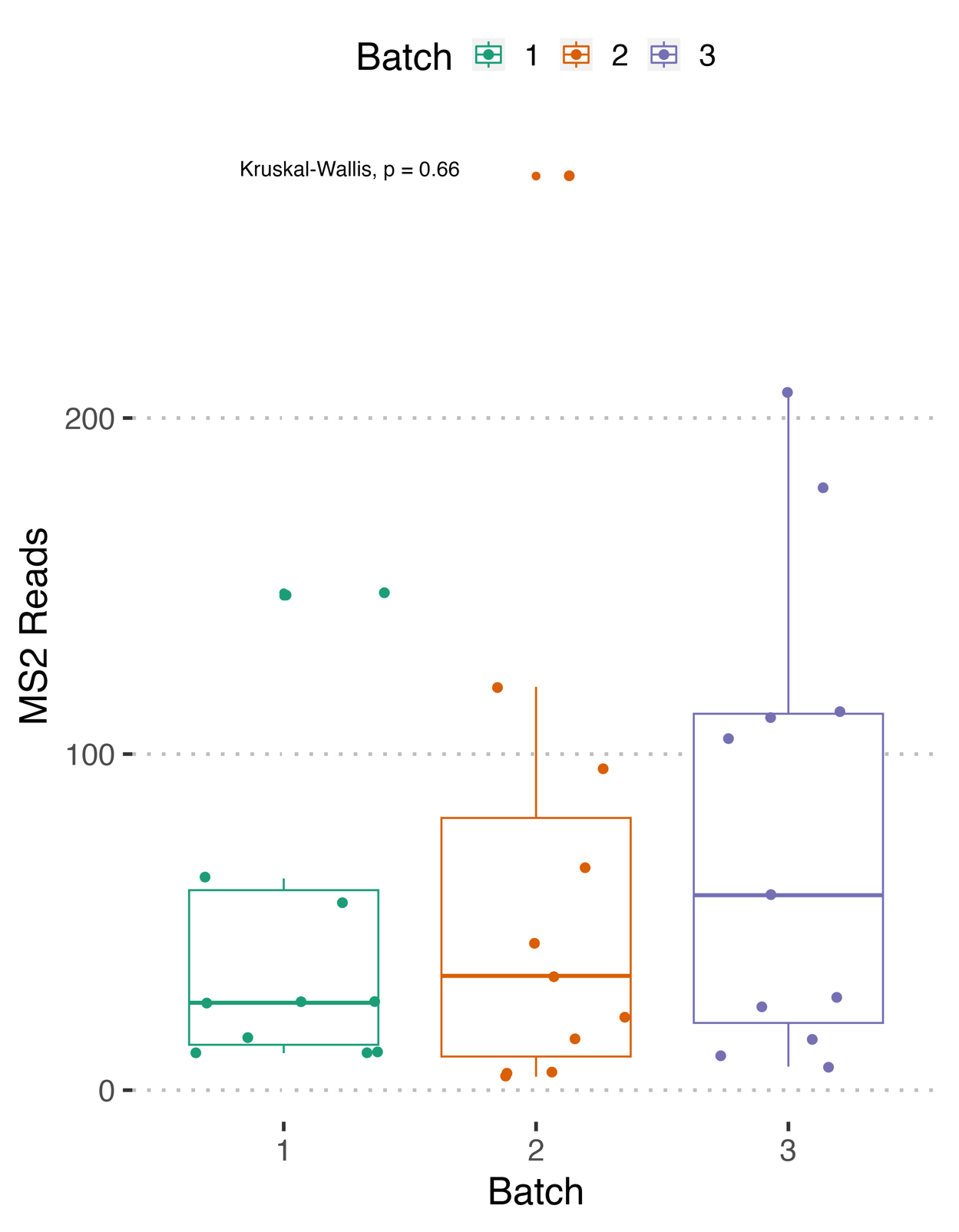


**Supplementary Figure 4.** Distribution of total MS2 bacteriophage reads for a set of eleven specimens that were sequenced on subsequent days (Batch 1-3), with different reagent lots, flowcells, and two operators. No significant difference in MS2 read distribution was observed.
