# Supplementary material for: Validation of an Automated, End-to-End Metagenomic Sequencing Assay for Agnostic Detection of Respiratory Viruses
Source: J Infect Dis. 2024 May 2;230(6):e1245–53. doi: 10.1093/infdis/jiae226 (PMC11646614; doi:10.1093/infdis/jiae226)
Supplement: jiae226_Supplementary_Data [file jiae226_supplementary_data.zip › Supplementary_Figure_5.docx]

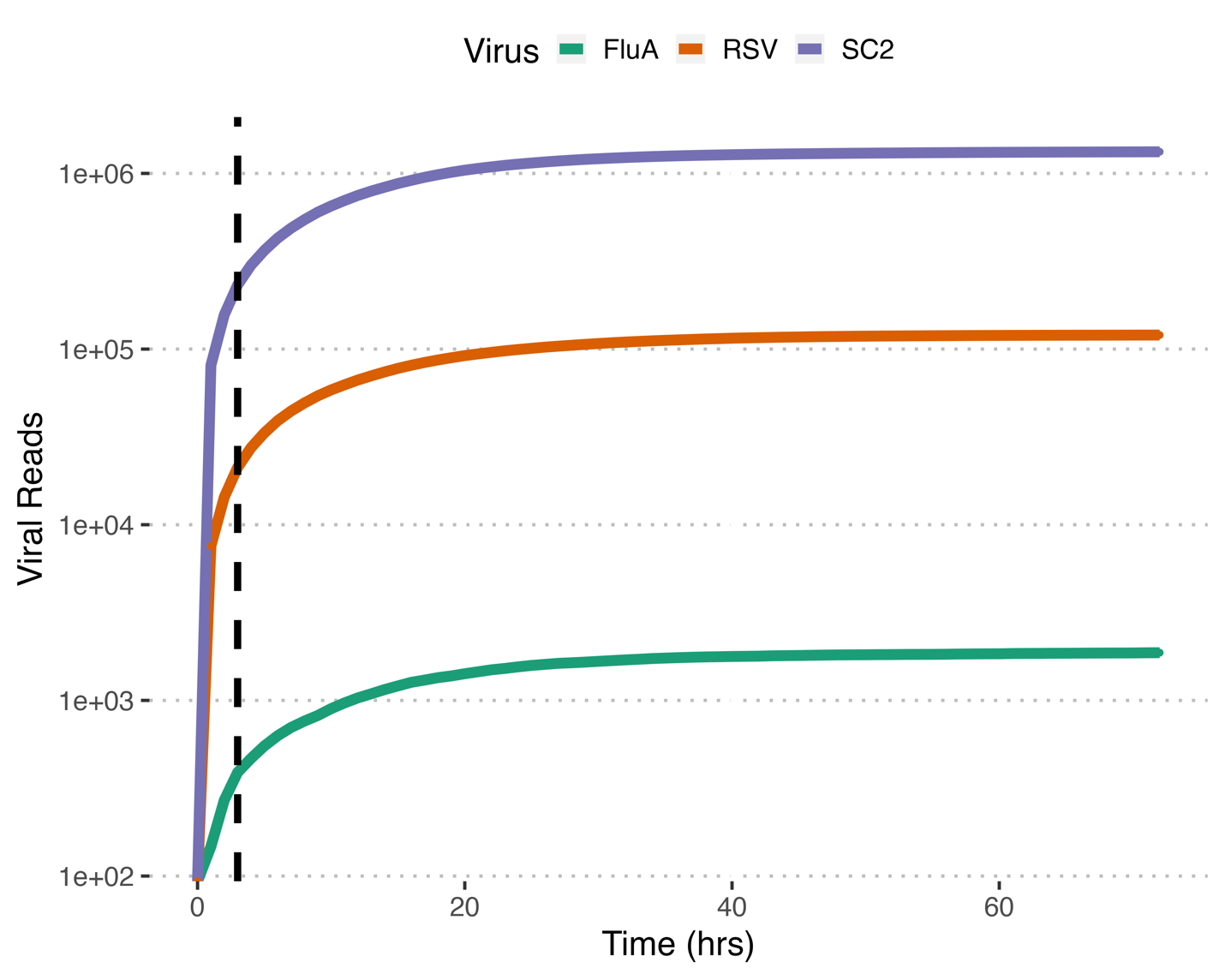


Supplementary Figure 5: Total viral read accumulation over time for SARS-CoV-2, RSV, and Influenza B RT-PCR positive specimens sequenced during clinical validation of the RAPID-mNGS assay. Dotted vertical line represents 3-hour reporting threshold.
