# Supplementary material for: Validation of an Automated, End-to-End Metagenomic Sequencing Assay for Agnostic Detection of Respiratory Viruses
Source: J Infect Dis. 2024 May 2;230(6):e1245–53. doi: 10.1093/infdis/jiae226 (PMC11646614; doi:10.1093/infdis/jiae226)
Supplement: jiae226_Supplementary_Data [file jiae226_supplementary_data.zip › Supplementary_Table_1.docx]

Supplementary Table 1: Summary of RT-PCR primer sequences and sources for quantification of viral loads.

| **Primer** | **Sequence (5’ to 3’)** | **Source** |
| --- | --- | --- |
| SARS-CoV-2 For | CTG CAG ATT TGG ATG ATT TCT CC | US CDC Flu/SC2 multiplex assay |
| SARS-CoV-2 Rev | CCT TGT GTG GTC TGC ATG AGT TTA G | US CDC Flu/SC2 multiplex assay |
| FluA For | CAA GAC CAA TCY TGT CAC CTC TGA C | US CDC Flu/SC2 multiplex assay |
| FluA Rev | CAA GAC CAA TYC TGT CAC CTY TGA C | US CDC Flu/SC2 multiplex assay |
| RNaseP For | AGA TTT GGA CCT GCG AGC G | US CDC Flu/SC2 multiplex assay |
| RNaseP Rev | GAG CGG CTG TCT CCA CAA GT | US CDC Flu/SC2 multiplex assay |
| RSV For | GGC AAA TAT GGA AAC ATA CGT GAA | Integrated DNA Technologies (Internal testing) |
| RSV Rev | TCT TTT TCT AGG ACA TTG TAY TGA ACA G | Integrated DNA Technologies (Internal testing) |
