# Supplementary material for: Validation of an Automated, End-to-End Metagenomic Sequencing Assay for Agnostic Detection of Respiratory Viruses
Source: J Infect Dis. 2024 May 2;230(6):e1245–53. doi: 10.1093/infdis/jiae226 (PMC11646614; doi:10.1093/infdis/jiae226)
Supplement: jiae226_Supplementary_Data [file jiae226_supplementary_data.zip › Supplementary_Table_2.docx]

Supplementary Table 2: Amplicon sequences for quantified oligonucleotides used to generate standard curves.

| **Target** | **Oligonucleotide sequence** | **Source** |
| --- | --- | --- |
| SARS-CoV-2 (N gene into intergenic) | 5'- ACC CGG CGC TAA CGC ACC TGC AGA TTT GGA TGA TTT CTC CAA ACA ATT GCA ACA ATC CAT GAG CAG TGC TGA CTC AAC TCA GGC CTA AAC TCA TGC AGA CCA CAC AAG GTC AAA CCC GGG GCC CGA -3' | IDT |
| RSV (M gene) | 5'- CTG AAG GAT GAG TGT CAG CCG GCA AAT ATG GAA ACA TAC GTG AAT AAA CTT CAC GAG GGC TCC ACA TAC ACA GCT GCT GTT CAA TAC AAT GTC CTA GAA AAA GAA GTG TAA CCC GAT GAG GTA CT -3' | IDT |
| FluA (Matrix 1) | 5’- TCT AAA CGC AAT GAG AGA GGT ATT CCT CAG GCC ACA TCG CTT CCT AGT TCC GCT GCA AGA CCA ATT CTG TCA CCT CTG ACT AAG GGG ATT TTG GGG TTT GTG TTC ACG CTC ACC GTG CCC AGT GAG CGA GGA CTG CAG CGT AGA CGC TTT GTC CAA AAT GCG GAT CCA TCG TTG GCG GCC GAA GCC GCC ATT CCA TAG TGA GTT CTT CGT C -3’ | Thermo Fisher GeneArt |
| RNaseP | 5’- GTA TAG TAT AGT GCG CTA ATC GGA GAC GAA TTT AAG ACA AGC AGA AGG TTT GAG GAT TAG GTC AAA TTG AAG ATT TGG ACC TGC GAG CGG GTT CTG ACC TGA AGG CTC TGC GCG GAC TTG TGG AGA CAG CCG CTC GTG GTT TAA TAT CGG TAT GTC TGG GAT TAA ACG AGT TCC CAA AAC CAA GCG GGC TCA CTA CGA CGG TTA A -3’ | Thermo Fisher GeneArt |
