# Supplementary material for: Validation of an Automated, End-to-End Metagenomic Sequencing Assay for Agnostic Detection of Respiratory Viruses
Source: J Infect Dis. 2024 May 2;230(6):e1245–53. doi: 10.1093/infdis/jiae226 (PMC11646614; doi:10.1093/infdis/jiae226)
Supplement: jiae226_Supplementary_Data [file jiae226_supplementary_data.zip › Supplementary_Table_3.docx]

Supplementary Table 3: Summary of SARS-CoV-2 cross contamination read percentages for three flowcells that contained at least one specimen with a high viral load (RT-PCR C_t_ < 15).

|  | **SARS-CoV-2 Reads Across All Samples (Total)** | **SARS-CoV-2 Reads in Negative Specimens (Contaminant)** | **Carryover/Crosstalk estimate (%)** |
| --- | --- | --- | --- |
| Flowcell 1 | 24652 | 2 | 0.0081 |
| Flowcell 2 | 58156 | 2 | 0.0034 |
| Flowcell 3 | 46390 | 7 | 0.015 |
