# Supplementary material for: Validation of an Automated, End-to-End Metagenomic Sequencing Assay for Agnostic Detection of Respiratory Viruses
Source: J Infect Dis. 2024 May 2;230(6):e1245–53. doi: 10.1093/infdis/jiae226 (PMC11646614; doi:10.1093/infdis/jiae226)
Supplement: jiae226_Supplementary_Data [file jiae226_supplementary_data.zip › Supplementary_Table_4.docx]

**Supplementary Table 4:** Subtype (Influenza A and RSV) and lineage (SARS-CoV-2) typing for RT-PCR positive specimens where viral reads were detected in RAPID-mNGS results. Data are summarized as the proportion of mNGS positive specimens where successful, as well as the number of specimens assigned to each strain.

| **Virus**  *Subtype/Lineage* | **Proportion of mNGS positive specimens** |
| --- | --- |
| **Influenza A**  *H1N1*  *H3N2* | 17/18 (94.4%)  5/17 (29.4%)  12/17 (70.6%) |
| **RSV**  *RSV A*  *RSV B* | 38/41 (92.5%)  20/38 (52.6%)  18/38 (47.4%) |
| **SARS-CoV-2**  *BA.5.2.1*  *BF.7*  *BQ.1.1*  *BQ.1.1.4*  *BQ.1.1.5*  *BQ.1.1.67*  *BQ.1.2*  *BQ.1.2.3*  *BY.1*  *DJ.1.1.1*  *XBB.1.5* | 20/37 (54.1%)  2/20 (10.0%)  1/20 (5.0%)  2/20 (10.0%)  1/20 (5.0%)  2/20 (10.0%)  2/20 (10.0%)  1/20 (5.0%)  4/20 (20.0%)  2/20 (10.0%)  1/20 (5.0%)  1/20 (5.0%) |
